# Bayesian multistate modelling of incomplete chronic disease burden data

**DOI:** 10.1093/jrsssa/qnac015

**Published:** 2023-01-24

**Authors:** Christopher Jackson, Belen Zapata-Diomedi, James Woodcock

**Affiliations:** MRC Biostatistics Unit, University of Cambridge; Healthy Liveable Cities Lab, Centre for Urban Research, RMIT University, Melbourne; MRC Epidemiology Unit, University of Cambridge

## Abstract

A widely-used model for determining the long-term health impacts of public health interventions, often called a “multistate lifetable”, requires estimates of incidence, case fatality, and sometimes also remission rates, for multiple diseases by age and gender. Generally, direct data on both incidence and case fatality are not available in every disease and setting. For example, we may know population mortality and prevalence rather than case fatality and incidence. This paper presents Bayesian continuous-time multistate models for estimating transition rates between disease states based on incomplete data. This builds on previous methods by using a formal statistical model with transparent data-generating assumptions, while providing accessible software as an R package. Rates for people of different ages and areas can be related flexibly through splines or hierarchical models. Previous methods are also extended to allow age-specific trends through calendar time. The model is used to estimate case fatality for multiple diseases in the city regions of England, based on incidence, prevalence and mortality data from the Global Burden of Disease study. The estimates can be used to inform health impact models relating to those diseases and areas. Different assumptions about rates are compared, and we check the influence of different data sources.

## 1 Introduction

**Figure 1:**
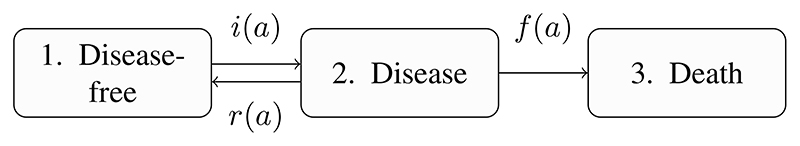
Multistate disease model with age-dependent incidence, case fatality and remission rates

To inform policies for chronic disease prevention, decision-makers need to quantify the expected impacts of interventions on population health. This requires knowledge of the current disease burden, e.g. incidence, prevalence, mortality, costs and inequalities, and how these outcomes might change under different scenarios or policies. Interventions affecting, e.g. diet, physical activity or air pollution exposure may affect multiple diseases in complex ways (Mytton et al., 2017; Briggs et al., 2019). Disease risks and the impacts of policies may also vary between different geographical areas, e.g. policies relating to transport (Iroz-Elardo et al., 2020). Since randomised trials for population health impacts are typically infeasible, theoretical or mechanistic models are required to enable simulation of how outcomes are generated and affected by the interventions (Threlfall et al., 2015). Different types of models used to simulate health and economic impacts of public health interventions to prevent chronic diseases are reviewed by Briggs et al. (2016). Many of these require knowledge of age-specific incidence, case fatality and remission rates for each disease of interest, so that each disease can be represented as a three-state transition model (Figure 1). This forms the basis of the “multistate lifetable” approach to impact modelling, or the “proportional multistate lifetable” (Barendregt et al., 1998; Blakely et al., 2020) approach if multiple diseases are modelled independently. Disease-related outcomes can then be simulated under different policies or scenarios that modify (at least) the disease incidence, either at an aggregate level for a homogenous population with common risks, or through individual-level “microsimulation” models that can represent large heterogeneous populations.

Data to inform those models could come from a range of sources, depending on the location and the detail required on how risks vary between people. Many diseases, such as cancer, have registries from which population incidence and case fatality can be estimated, however information for subgroups of the population may be restricted. Commonly, indirect evidence is required. For example, annual risks of mortality from a disease, for a population including people with and without that disease, are often published by national authorities. However, case fatality, the risk of death for people with the disease, is less commonly available. Age-specific prevalence might also be obtained from survey data, and used to infer disease incidence (Keiding, 1991). The Global Burden of Disease (GBD) project (GBD 2019 Diseases and Injuries Collaborators, 2020) publishes estimates of incidence, prevalence and mortality, but not case fatality, for hundreds of diseases, countries and sub-national regions worldwide. This is a convenient source to inform health impact models that involve many diseases and are designed to be easily adaptable to different geographical settings. An example is the “Integrated Transport and Health Impact Model” (ITHIM) series of models used to predict the effects of changes in transport behaviours and/policies in different settings (Woodcock et al., 2014; de Sá et al., 2017; Jaller et al., 2020).

Many models to estimate the health impacts of disease prevention interventions (e.g. Rehm et al., 2009; Cecchini et al., 2010; Kypridemos et al., 2016; Mytton et al., 2018) have used the DisMod II method and software (Barendregt et al., 2003), to estimate the quantities in the model in Figure 1 given a mixture of direct and/or indirect (prevalence and mortality) data. This method estimates the unknown incidence, case fatality and remission rates in Figure 1 through optimising a squared error loss. In Section 2.2 we clarify the statistical principles behind this method, which have not previously been made explicit. Essentially, it is a maximum likelihood procedure, however it makes strong assumptions about homogeneity of error variances between ages, and uses some ad-hoc procedures with unclear statistical properties, e.g. to produce rates as smooth functions of age (see Section 2.4). The assumption that trends through time are common to all ages has been shown to be unsuitable in particular situations (Scarborough et al., 2016). The software is available as a interactive Windows interface, though analysis settings must be specifed with “point and click”, which makes reproducibility difficult.

An alternative approach to the same estimation problem was described by Flaxman et al. (2015). This is based on an explicit sampling model for the observed data that represents all structural assumptions, e.g. about how risks are related between different people. Uncertainties are quantified through Bayesian inference, and the model is fitted by Markov Chain Monte Carlo (MCMC) sampling. Risks depend on age through linear spline functions, though sensitivity to the choice of the spline knots and smoothness parameter was highlighted. An empirical Bayes approach was used to share information between areas, by modelling each region separately using a prior obtained from worldwide data. While Python code to implement models of this form has been published (as DisMod-MR, Flaxman, 2019), the details of the models implemented in this code, and the quantities that can be estimated from them, are not fully documented, and we are not aware of any applications of the method outside the Global Burden of Disease project.

We develop a modelling framework and software package which combines the statistical formality of the Flaxman et al. (2015) methods with the accessibility of the DisMod II tool. A Bayesian model is used, with two alternative computational methods. The more accurate, but more expensive, method uses Markov Chain Monte Carlo to sample from the full posterior distribution. Alternatively, the posterior mode can be determined using optimisation, and supplemented with an approximation to the posterior. That allows an exact point estimate to be produced, and uncertainty to be quantified, without computational expense. Our models extend those described by Flaxman et al. (2015) in the following ways. To represent variation in risks with age, instead of a linear spline with pre-specified smoothness, a penalised smooth spline is used that allows the appropriate amount of smoothness to be determined from the data. To represent variation in risks between areas, as well as empirical Bayes methods, a full hierarchical model can be used which additionally accounts for uncertainty about between-area variations in risk. The model is also extended to allow risks to depend on calendar time as well as age, while relaxing DisMod II’s assumption that the same time trend applies to all ages. More precise estimates can also be produced in situations where the effect of a predictor (typically gender) can be assumed to be common between areas. Structural assumptions, such as constant risks under a certain age, can be employed to strengthen inferences from sparse data, and alternative models can be compared against observed data by cross-validation. The methods are made available in a thoroughly-documented R package at http://chjackson.github.io/disbayes.

The Bayesian model, and how it is estimated from data, is fully explained in Section 2. The methods we develop are motivated by the demands of health impact modelling in the city regions (or “combined authority” areas) of England, described further in Section 3. Published estimates and uncertainty intervals for age-specific incidence, prevalence and mortality for the relevant local areas are obtained from the Global Burden of Disease project. From these data, our Bayesian model enables case fatality rates to be inferred alongside incidence rates. This produces a database of estimated disease transition rates that can be used for any multistate lifetable health impact model relating to these diseases and areas. We compare the plausibility and statistical fit of different model assumptions, and discuss the influence of different sources of data on the results. Section 4 concludes the paper with a discussion of further challenges of multistate disease modelling for both disease burden estimation and health impact estimation.

## 2 Models

### 2.1 Theoretical disease model

We represent a disease as a continuous-time, multistate process with three states: (1) disease-free, (2) disease, (3) death from the disease (Figure 1). The individual-level disease process is fully defined by the disease incidence *i*(*a*), the remission rate *r*(*a*) and the case fatality rate *f*(*a*), which are assumed to depend on age *a*, defining a continuous-time, age-inhomogeneous Markov model. For some diseases, remission might be assumed to be implausible, so that *r*(*a*) = 0; an equivalent assumption is that the case fatality from a disease is constant for all times since first onset of the disease until death. Mortality from other causes is assumed to be independent of disease status, so that the death state for each disease represents only deaths caused by that disease, ignoring other causes, and the rates pertaining to different diseases are estimated independently of each other (Barendregt et al., 1998).

Assume further that these rates are constant within integer years of age *a*, so they can be written *i_a_*, *f_a_*, *r_a_*. For the moment, assume also that the rates do not vary with calendar year, though this assumption is unrealistic for some diseases and will be relaxed later. Various quantities of interest can be defined as functions of these, as follows.

- The annual *transition probability matrix P_a_*, whose *r*, *s* entry *P*_*a*,*r*,*s*_ is the probability that a person is in state *s* at age *a* + 1 given they are in state *r* at age *a*. *P_a_* is a deterministic function of *i_a_*, *f_a_* and *r_a_*, which is the solution to a differential equation, given in full in the supplementary Appendix.
- The *state occupancy probabilities S_a_* or the proportion of individuals in a hypothetical birth cohort (of infinite size) who are in each state at age *a*. This is a vector with three elements *S_aj_*, one for each state *j*. The disease prevalence at age zero is fixed, typically so that everyone is disease-free at age 0. The state occupancy probabilities at each subsequent age *a* + 1 can then be determined by multiplying by the transition probability matrix:
  $$S_{a+1}=S_{a}P_{a}$$
- The *prevalence* of disease among people who are alive at each age *a* (in the infinite population):
  (1) $$\pi_{a}=S_{a,2}/(S_{a,1}+S_{a,2})$$
- The population disease-specific *mortality probability d_a_* at age *a*, or the probability that a person alive at age *a* dies from the disease before age *a* + 1, which is a function of the disease prevalence *π_α_* at age *a* and the transition probabilities *P_a_* between ages *a* and *a* + 1,
  (2) $$d_{a}=P_{a23}\pi_{a}+P_{a13}(1-\pi_{a})$$

To start with, suppose we are modelling a homogeneous population (e.g. as defined by gender and area) where the rates vary only by age. In Sections 2.2–2.3 we discuss methods for estimating unknown quantities in this model from data, and in Section 2.4 we explain how the dependence on age can be modelled flexibly and efficiently. In Section 2.5 the model will be extended to represent populations from different areas that are related through a hierarchical model, and Section 2.6 describes how rates can be related parsimoniously between different subgroups, e.g. by gender. Section 2.7 then explains how trends in risks through calendar time can be modelled.

### 2.2 Approaches to inference from data

We assume there are data on mortality from the disease, and at least one of incidence or prevalence, from years of age *a* = 0,…,*A*. If remission is permitted, there may also be similar data on those assumed to be cured of the disease. To motivate how we estimate the model in Section 2.1 from data, first we explain how the commonly-used DisMod II method (Barendregt et al., 2003) works.

In DisMod II, the input disease data are provided as point estimates, e.g. of prevalence or mortality. The unknown transition rates *i_a_*,*f_a_*,*r_a_* are then estimated by minimising the sum of squared errors between these estimates and those implied by the theoretical model of Section 2.1. The statistical theory behind this procedure is not made explicit, but it is equivalent to maximum likelihood estimation, under the assumption that the point estimates are observations from a normal sampling distribution with mean defined by the true quantity, and a variance that is the same for each age. In reality, however, the error variance will be greater for ages at which the input quantities are more uncertain due to being obtained from smaller samples (e.g. because the population at risk varies with age).

Uncertainty in DisMod II can be quantified by supplying distributions around the inputs. Monte Carlo simulation from these distributions is then used to determine the implied distribution of the estimated transition rates. Therefore, if the user supplies the correct sampling distributions behind their input data, this is equivalent to a bootstrap method to estimate the sampling distribution of the transition rate estimators. No guidance is given, however, on how a user can derive those input distributions to reflect the knowledge that is available in real situations. As well as knowledge about sampling variability, this includes “structural” knowledge about how rates for different people (e.g. of different ages, genders and areas) are expected to be related to each other, and expected biases or inconsistencies between data sources. In DisMod II, structural knowledge can be introduced by ad-hoc methods, e.g. by smoothing age-specific point estimates after estimation to reflect that rates are expected to be similar between ages, though, as discussed in Section 2.4, this does not use all information efficiently.

Therefore, instead of expressing the problem as minimising a loss, we prefer to define a statistical model from which we assume the data are generated, and where all data-generating assumptions are made explicit. This produces a likelihood function for the unknown parameters, which can either be maximised or combined with a prior in Bayesian inference. Uncertainties can then be automatically represented, either through obtaining the covariance matrix of the maximum likelihood estimates, or in the posterior distribution in the Bayesian approach. As in Flaxman et al. (2015), we use Bayesian inference, and any relations between rates from different people are explicitly described in the model or as prior distributions. The details of this model, and how we extend on the Flaxman et al. (2015) method, are described in the following sections.

### 2.3 Binomial likelihood for a homogeneous population

Instead of point estimates, the data are expressed as counts, which explicitly acknowledges that they were obtained from observing disease outcomes from a finite population. Specifically, we suppose we have numerators and denominators, as follows.

incidence: given a population at risk, of size $n_{a}^{\left(inc\right)}$, $y_{a}^{\left(inc\right)}$ of these are observed to get the disease within the next year, mortality: given a population at risk, of size $n_{a}^{\left(mort\right)}$ (with or without the disease), $y_{a}^{\left(mort\right)}$ of these are observed to die from the disease within the next year,

prevalence: from a sample of $n_{a}^{\left(prev\right)}$ individuals, $y_{a}^{\left(\text{prev }\right)}$ are known to have the disease, and $n_{a}^{\left(prev\right)}-y_{a}^{\left(\text{prev }\right)}$ are known to not have the disease, at age *a*.

Remission data $y_{a}^{\left(rem\right)}$, $n_{a}^{\left(rem\right)}$ may also be available in a similar form, if remission is permitted.

As shown in Appendix Section B, if the data are published as point estimates, with a (published or assumed) measure of uncertainty around the estimates, we can derive approximately equivalent numerators and denominators of the required form. Furthermore, the data may be published as estimates for coarser age groups (5 or 10 year bands) while estimates are required for one year of age. A procedure for smoothly disaggregating counts for age groups into estimated year-specific counts, with no loss of information, is discussed in Appendix Section C.

Under the theoretical disease model, if individuals in the population are assumed to be independent with identical risks, these data are generated as

incidence: $y_{a}^{\left(\text{inc }\right)}∼Binomial\left(n_{a},1-P_{a11}\right)$, where 1 – *P*_*a*11_ is the probability of getting the disease at some time within a year, which we will call the *incidence probability*.

mortality: $y_{a}^{\left(\text{mort }\right)}∼Binomial\left(n_{a},d_{a}\right)$, where the disease-specific mortality *d_a_* is a deterministic function of the incidences and case fatalities {*i_j_*, *f_j_*} for ages *j* up to *a* (Equation 2).

prevalence: $y_{a}^{\left(\text{prev }\right)}∼Binomial(n_{a}^{\left(\text{prev }\right)},\pi_{a})$, where *π_a_* is the theoretical prevalence, defined as a deterministic function of the incidences and case fatalities (Equation 1).

remission: $y_{a}^{\left(\text{rem }\right)}∼Binomial(n_{a}^{\left(\text{rem }\right)},P_{a,2,1})$, where *P*_*a*,2,1_ is the annual transition probability from disease to health.

The Binomial model above defines a full likelihood *L*(*θ*|y) for the data $y=\left\{y_{a}^{\left(mort\right)},y_{a}^{\left(\text{prev }\right)},y_{a}^{\left(\text{inc }\right)},y_{a}^{\left(\text{rem }\right)}\right\}:a=0,\ldots ,A$ and parameters given by the rates *θ* = {*i_a_*, *f_a_*,*y_a_*} : *a* = 0,…,*A*.

To estimate the rates, in theory, this might be maximised as a function of the *θ*, independently for each population that we want to describe (e.g. by area and gender). Similarly, Bayesian inference might be used with independent priors for the rates at each year of age *a*. However, this ignores the knowledge that risks at adjacent ages will be similar, and the data will often be too weak to allow all of the age-specific parameters to be identified solely from age-specific data — in particular, the case fatality rates *f_a_* will be too weakly informed by the data on mortality, in settings and ages where the disease is uncommon.

Instead, we build in structural assumptions that will define how rates for particular people are assumed to be similar to each other. Firstly, Section 2.4 defines a model for how rates depend on age. Later we will define models that can be used to describe how rates vary between different contexts such as geographical areas (Section 2.5) or by gender (Section 2.6). Building in plausible structural relations between parts of the data in this way improves identifiability of the estimates and increases their precision. Rates may also vary through calendar time (Section 2.7). A further challenge is that the datasets informing incidence, mortality, prevalence and remission might be obtained from different sources, hence describe populations with slightly different epidemiology (Section 2.8).

### 2.4 Spline models for dependence on age

Since the rates for similar ages are expected to be similar, the case fatality and incidence are assumed to be smooth, nonlinear functions of age. The model for case fatality is:

(3) $$\log\left(f_{a}\right)=\overset{k}{\underset{k=1}{\sum^{\text{​}}}}\beta_{k}g_{k}(a)$$

where *g_k_*() are spline basis functions. The models for incidence and remission rates have an identical form, but governed by different parameters.

Any spline basis can be used in theory. Flaxman et al. (2015) describe linear splines with pre-specified knots and smoothness penalties. We extend this by allowing the appropriate shape and smoothness to be estimated from the data as part of the model fit, and we use a more flexible “thin plate” spline, parameterised as in Wood (2016) to facilitate Bayesian modelling. The exact form of this basis is complex, and described in full in Wood (2017). A large number *K* = 10 of basis terms are included to ensure high flexibility if required. Note this does not necessarily give an over-parameterised model, because the appropriate level of flexibility is estimated, as we now describe. The first two terms represent an intercept and slope, *g*_1_(*a*) = 1, *g*_2_(*a*) = *a*. The remaining terms represent departures from a linear relationship of the log rate with age, and their coefficients are assumed to be exchangeable draws from a common distribution $\beta_{k}∼N(0,\lambda_{0}^{2})$ for *k* ≥ 3. The prior standard deviation λ_0_ controls the degree of smoothness, with the curve tending towards a straight line for λ_0_ → 0 and becoming more flexible for large λ_0_. A prior is placed on λ_0_ (we use a Gamma(2,1) to facilitate estimation of the posterior mode, see Gelman et al. (2013), Chapter 10) hence Bayesian updating of this parameter allows the appropriate amount of smoothness to be estimated from the data.

If we are modelling a homogeneous population (e.g. a specific area and gender) we can then complete the model by defining priors for the intercept *β*_1_ and slope *β*_2_. In the application, we use vague *N*(0,100^2^) priors.

The parameters *β_k_* and λ_0_ defining the age curve are included in the likelihood and estimated as part of the Bayesian model. This differs from Dismod Il’s approach to smoothing, where age-specific rates are estimated independently, and the resulting estimates can then be smoothed. While that ensures that estimates from adjacent ages are similar, an advantage of our approach, where the rates and the functional form relating them are estimated in a single step, is that it allows the improvements in precision from borrowing information between ages to be accounted for in reduced uncertainty around the estimates.

Two further assumptions about age dependence of disease rates are used, depending on the disease. Firstly, we define an age *a_base_* below which rates are assumed to be constant. For example, incidence may be zero or low for some diseases at younger ages. Or relatedly, if the prevalence of the disease is low at younger ages, then there will be no information about case fatality at these ages, meaning that the model needs to constrain how case fatality depends on age. Secondly, for some diseases, we impose the additional constraint that the rates are *increasing* with age (after the period when rates are constant). This is enabled by using the same form of spline model, but for the log *increments* in rates with each year of age, rather than the log rates.

An alternative model with independent vague priors for each year of age was also found to be useful for model development — since if the age-specific posterior for case fatality is identical to the age-specific prior in that model, we can deduce that the data provide no information about that particular age, confirming that additional structural or prior assumptions would be needed instead.

### 2.5 Hierarchical model for variations between areas

For some diseases and areas, the information in a single area alone may be too weak to give sufficiently precise estimates of case fatality or incidence. However, a single area’s data can be strengthened through the information provided by other areas. As an alternative to aggregating the data over areas, a hierarchical model can be used, which “partially pools” weak data from one area with the average from other areas. In DisMod II there is no hierarchical modelling ability. Flaxman et al. (2015) implemented partial pooling by using estimates from data aggregated over multiple areas to define a prior for the area-specific rate in models fitted to area-specific datasets independently. They also investigated a full Bayesian hierarchical model, which represents between-area variations as random effects and has the advantage of fully accounting for uncertainties about between-area variations, but found this model to be computationally prohibitive.

Here we describe a similar fully Bayesian hierarchical model, and implement it using Hamiltonian Monte Carlo methods (see Section 2.9), which are designed to explore correlated posterior distributions more efficiently than the Metropolis-Hastings methods that were used by Flaxman et al. (2015). In our applications, convergence, and 1000 uncorrelated samples from the posterior, were achieved in 1-5 hours of running time, depending on the structural assumptions. We also implement an efficient approximation to the full hierarchical model (Section 2.9) that ran within minutes in our examples.

In this setting, there are data $y=\left\{y_{i,a}^{\left(\text{mort }\right)},y_{i,a}^{\left(\text{prev }\right)},y_{i,a}^{\left(\text{inc }\right)}\right\}$, published by area *i* as well as age *a*, and are related to area-specific rates *θ* = {*i*_*i*,*a*_, *f*_*i*,*a*_, *y*_*i*,*a*_} through the model described in Sections 2.1–2.3, leading to a joint likelihood *L*(*θ*|y) over ages and areas. As before, the rates are smooth spline functions of age, and the models for incidence and case fatality have the same structure, but with different parameters for each area. For case fatality, $\log\left(f_{i,a}\right)=\sum_{k=1}^{K}\beta_{i,k}g_{k}(a)$, and for incidence, $\log\left(i_{i,a}\right)=\sum_{k=1}^{K}\beta_{i,k}^{\left(inc\right)}g_{k}(a)$. Remission rates might be treated in the same way in principle, but given the available data in the application, these are assumed to be constant over areas as well as ages. Then to make this model hierarchical, the intercept term *β*_1_ in the spline function becomes a *random effect β*_*i*,1_, and is given a distribution that represents the variation between areas in the level of risk. In the application in Section 3, the random intercepts for log case fatality are assumed to be exchangeable, $\beta_{i,1}∼N(b_{1},\lambda_{1}^{2})$. A random slope could be defined similarly by placing a distribution on *β*_*i*,2_, however in the application this was judged to be unnecessary, and a common slope *β*_*i*,2_ = *b*_2_ was used.

The sharing of information can be controlled by the prior distribution for the between-area random effect standard deviation λ_1_. To allow this prior to be defined transparently, we imagine “high-risk” and “low-risk” areas, defined by the 2.5% and 97.5% quantile of the distribution of case fatality, and place a prior guess on the ratio in case fatality between a high and low risk area, and a plausible upper limit on this ratio. A guess of a 5-fold ratio, with an upper limit of a 50-fold ratio, is used later in the application. A gamma prior distribution for λ_1_ is then obtained by a numerical search for the gamma shape and scale parameters that correspond to this prior mean and 97.5% upper quantile. The nonlinear terms *β*_*i*3_,…, *β*_*i,K*_ are given identical $N(0,\lambda_{0}^{2})$ priors, where λ_0_ again controls the degree of smoothness, which is assumed to be the same for all areas *i*. A vague *N*(0,10^2^) prior is used for the mean intercept, *b*_1_, and a *N*(5, 5^2^) for the common slope *b*_2_ – more informative than the priors for *β*_1_, *β*_2_ used in 2.4, to facilitate computation, though still covering an extremely wide range.

### 2.6 HIerarchical models with additive gender and area effects

Typically we will want to estimate how disease risks differ by gender as well as age, and there will be data by age a, area *i* and gender *j*, e.g. outcome counts $y=\left\{y_{i,a\cdot j}^{\left(\text{mort }\right)},y_{i,a.j}^{\left(\text{prev }\right)},y_{i,a\cdot j}^{\left(\text{inc }\right)}\right\}:a=1,\ldots A;j=1,2$ and corresponding denominators. The data for a specific *i*, *j* are again assumed to be generated from the same theoretical disease model, with parameters given by the rates $\theta =\left\{i_{i,a.j},f_{i,a,j},y_{i,a,j}\right\}:a=1,\ldots ,A$. Instead of analysing the datasets for each gender independently under the previously-described models, more precise estimates might be produced under an assumption that the effect of gender is *independent of the effect of the area*. Then, after adding a third index *j* in the model for case fatality: $\log(f_{i,a,j})=\sum_{k=1}^{K}\beta_{i,j,k}g_{k}(a),\log(i_{i,a,j})\;=\sum_{k=1}^{K}\beta_{i,j,k}g_{k}(a)$, we would have $\beta_{i,j,k}=\beta_{i,k}^{\left(\text{area }\right)}$ for females (*j* = 1), and $\beta_{i,j,k}=\beta_{i,k}^{\left(\text{area }\right)}+\beta_{k}^{\left(\text{male }\right)}$ for males (*j* = 2).

$\beta_{i,k}^{\left(\text{area }\right)}$ are assigned the same priors as the *β*_*i*,*k*_ in Section 2.5. In our case study, a normal prior with mean 0 and standard deviation 0.82 is used for $\beta_{1}^{\left(\text{male }\right)}$ and $\beta_{2}^{\left(\text{male }\right)}$, the gender effect on intercepts and slopes of the log-linear model, representing a 95% prior probability that the female/male rate ratio, and the female/male ratio in age trends, are between 0.2 and 5. For *k* > 2, the effects $\beta_{k}^{\left(male\right)}$ of gender on each of the *k*th spline basis coefficients are given $N(0,\lambda_{0}^{{\left(\text{male }\right)}^{2}})$ priors, enabling the gender effect to deviate flexibly from a linear function of age. $\lambda_{0}^{\left(\text{male }\right)}$ determines the flexibility of this function, and can be fixed or given a prior and estimated.

### 2.7 Time trends

The model presented so far, specifically the recursive definition in Section 2.1 of the state occupancy probabilities *S_a_* and prevalence at age *a* in terms of risks at all previous ages, assumes that risks for a particular age *a* do not change through calendar time. Therefore, for example, when using data for a mixed population from one year (2017 say), the model assumes that the risk faced by a person of someone of age 50 in 2017 is the same as the risk faced in 1997 by someone aged 70 in 2017 (who were 50 in 1997). This is unrealistic for many diseases and populations. From data by age group for one calendar year, it is not possible to distinguish trends through time from differences between age groups. In theory, these might be distinguished by extending the framework in Section 2.3 to include cross-sectional data from multiple years, however, we expect that identifiability and sampling from the posterior distribution would be difficult.

Therefore, instead of using our model to *estimate* time trends from data, we extend the model to include published point estimates of time trends as constants, to determinine the *consequences* of these trends for our inference of current incidence and case fatality from current data. The rates *i_a_*, *f_a_*, *r_a_* now describe risks in the current year, that is the year represented by the data in Section 2.3. Risks in previous years are defined by multiplying the current risks by a constant ratio determined from literature on disease trends. This allows the model to be extended so that incidence and case fatality depend on both age and (calendar) year, $i_{a,y}=\rho_{a,y}^{\left(i\right)}i_{a}$, $f_{a,y}=\rho_{a,y}^{\left(f\right)}f_{a}$, where the *ρ* indicate the ratios determined from literature, assumed to be known perfectly, and the year of the data is labelled *y* = 100, thus the earliest year represented is 100 years prior to this, *y* = 0, the year of birth for people 100 years of age in the current data. The models in DisMod II allowed time trends in a similar way, but the risk ratios *ρ*_*a*,*y*_ were restricted to be the same for all ages *a*, while our model allows them to be age-dependent. The models in Flaxman et al. (2015) assumed no time trends.

The age and year-dependent rates can then be used to define age and year-dependent transition probabilites and state occupancy probabilities *P*_*a*,*y*_, *S*_*a*,*y*_ that are assumed to generate the observed data. The data are the same as before — note that they are only available for year *y* = 100. The mortality data, for example, follows the same binomial model defined in Section 2.3. The probability *d_a_* governing this model, however, is now defined in terms of age and year-specific transition probabilities *P*_*a*,*y*,*r*,*s*_, and the prevalence *π*_*a*,*y*_ = *S*_*a*,*y*,2_/(*S*_*a*,*y*,1_ + *S*_*a*,*y*,2_) at *y* = 100. For each age *a*,

$$d_{a}=P_{a,y=100,2,3}\pi_{a,y=100}+P_{a,y=100,1,3}(1-\pi_{a,y=100})$$

*P*_*a*,*y*,*r*,*s*_ is the probability that a person is in state *s* at age *a* +1 and year *y* + 1 given they are in state *r* at age *a* and year *y*. The matrix *P*_*a*,*y*_ is defined as a function of *i*_*a*,*y*_ and *f*_*a*,*y*_, the same function used to define the year-independent matrix. *S*_*a*,*y*_ is the vector of probabilities that a person born in year *y* – *a* (thus is of age *a* at year *y*) occupies each of the states at year y, again defined recursively as *S*_*a*,*y*_ = *S*_*a*−1,*y*−1_*P*_*a*−1,*y*−1_, where *S*_0,*y*_ is fixed, e.g so that people are disease-free at age 0 with probability 1, and we need to know *P_b,y_* for each pair of (*b*, *y*) = (*a* – 1, 99), (*a* – 2, 98),…, (0,100 – a), for each of *a* = 1,…, 100.

This approach is illustrated in Section 3 for ischemic heart disease.

### 2.8 Model checking and comparison

The model in section 2.3 assumes that the disease rates that generate the three or four different data sources (incidence, prevalence, mortality and remission) are the same. In reality, the data on these sources may have been obtained from different populations, perhaps at different times or places, with different disease epidemiology. Using the model in that case will produce “average” parameter estimates which describe an unclearly-defined “mixed” population. Ideally, the inferences from the model should have a transparent connection to a dataset that describes a known population.

Consistency between data sources can be checked by comparing observed data with corresponding estimates obtained from the model. For example, suppose there is direct data on incidence, and the model is used to jointly estimate incidence and fatality rates from mortality, prevalence and incidence data. We could compare observed incidence data with incidence estimates from the model, and if they disagree to an extent that is unexplainable by sampling variation, then this suggests that the prevalence and mortality data provide evidence about incidence that conflicts with the direct data on incidence. Conflicts might occur if time trends in incidence have not been properly accounted for, so that past incidence (which generated the current prevalence and mortality data) is different from current incidence, or if the underlying populations behind the data sources are otherwise different.

Biases might be handled by better adjustment for time trends, by excluding the biased data source if it is not necessary, or by adding model parameters that describe the differences between the data sources (akin to the “node splitting” ideas of Presanis et al. (2013)). However there is a limit to how much we can accommodate biases without making it impossible to learn from the data, e.g. information about both mortality and prevalence is required to be able to estimate case fatality.

To compare different models for the same data, e.g. a simpler or more constrained model (that might be biased) and a more complex or flexible model (that may be estimated imprecisely), their predictive ability can be assessed using leave-one-out cross-validation, via the method and R package of Vehtari et al. (2017, 2020). For each observation *i*, this method estimates *elpd_i_*, the expected log predictive density (ELPD), a measure of the accuracy with which a model would predict the *i*th observation if it were left out when fitting the model. In our application, the ith observation is defined as the outcome count $y_{a}^{\left(out\right)}$ (Section 2.3) for the ith combination of age and outcome type *out* (incidence, mortality, prevalence or remission), in addition to area or gender if modelled. The sum Σ_*i*_ *elpdi_i_*, over all *i* for some outcome, gives an overall measure of the accuracy with which a model predicts that outcome.

### 2.9 Computation and software

The joint likelihoods and priors are fully defined by the data model from Section 2.3 together with the age dependence model (Section 3), and potentially also the models to combine data from different areas (Section 2.5) or genders (Section 2.6), or to account for time trends (Section 2.7). In each case, the posterior requires simulation to describe, which is done here using two alternative methods. The more expensive but more accurate approach is to sample from the exact posterior by a Markov Chain Monte Carlo algorithm, similarly to Flaxman et al. (2015). A faster alternative is to use numerical optimisation to find the mode of the posterior. When the mode is found, a sample can be produced instantly from a multivariate normal approximation to the posterior, defined through a second-order approximation to the log density function on the unconstrained parameter space (see Gelman et al. (2013), Chapter 4). In these models, the normal approximation can be computed practically instantly, at around 1/100 of the running time of the equivalent MCMC procedure. The optimisation method is just as fast as the optimisation used by DisMod II, with the advantage of being based on a explicit statistical model, as described in 2.2. A further advantage is that approximate uncertainty intervals are produced more efficiently than in Dismod II’s procedure of repeatedly computing estimated rates for each resampled input dataset.

An R package disbayes was developed, which implements any of the models described here, using the Hamiltonian MCMC and optimisation procedures available in the Stan software, used through its rstan interface (Stan Development Team, 2020). This package is available from http://chjackson.github.io/disbayes, with thorough documentation and worked examples, and is described in more detail in Appendix Section D.

## 3 Application: estimating multistate lifetable data for city regions in England

We aim to estimate disease transition rates that can inform health impact models for use in the city regions of England. These include models for changes in active transport, that build on the “Integrated Transport and Health Impact Model” series of models (from Woodcock et al., 2014; de Sá et al., 2017; Jaller et al., 2020). Multi-state lifetable models are required to simulate long-term population disease outcomes under scenarios where incidence and case fatality are modified by changes in risk, e.g. due to increased physical activity. This requires area-specific estimates of incidence and case fatality of multiple chronic diseases that may be affected by changes in the relevant risk factors. While direct data on incidence is available for the required diseases and areas, case fatality is not. In this section, we show how our model is used to infer case fatality, along with incidence, from data on prevalence, incidence and mortality and remission. Full code and data to reproduce the analyses in this section is available at http://github.com/chjackson/disbayes, in the metahit folder.

### 3.1 Disease data

Data on incidence, prevalence, and mortality for chronic diseases in the year 2019 are obtained from the Global Burden of Disease (GBD) project (GBD 2019 Diseases and Injuries Collaborators, 2020) (URL: http://ghdx.healthdata.org/gbd-2019) by gender, 5-year age groups (from 0-4 to 95-99) and local authority districts within 9 English city regions. The chronic diseases included are those with evidence of an association with physical activity, air pollution or noise exposure. For concise presentation, in this paper we illustrate the analyses for the six of these that are associated with the greatest numbers of deaths (ischemic heart disease, stroke, chronic obstructive pulmonary disease, lung cancer, colorectal cancer, breast cancer and dementia), and two less common diseases (stomach and uterine cancer). Seven additional diseases are included in the full analysis available online.

The published data consist of estimated probabilities with 95% credible intervals. These were converted to an approximate numerator and denominator, using the procedure described in Appendix Section B. Those counts, for 5-year age ranges, are then disaggregated to give smoothly-varying 1-year counts, using the methods implemented by Sax and Steiner (2013). This results in one-year counts that are constrained to sum to the five-year totals, but vary smoothly by age. These counts are then aggregated over local authority districts within each city region (as defined in https://www.ons.gov.uk/economy/economicoutputandproductivity/output/articles/cityregionsarticle/2015-07-24#appendix), to produce counts for a city region.

Information about remission rates for each of the cancers is obtained from published estimates and confidence intervals for 10-year survival probabilities for England, published by age ranges (of width 10 years or more) (Office for National Statistics, 2019). As in the GBD study (GBD 2019 Diseases and Injuries Collaborators, 2020), we assume that 10-year survival implies that remission happened within those 10 years. An annual remission probability *r* can then be deduced from the 10-year survival probability 1 – (1 – *r*)^10^. The probabilities are converted to numerators and denominators, disaggregated to years of age, and assumed to be equal for all city regions.

**Figure 2:**
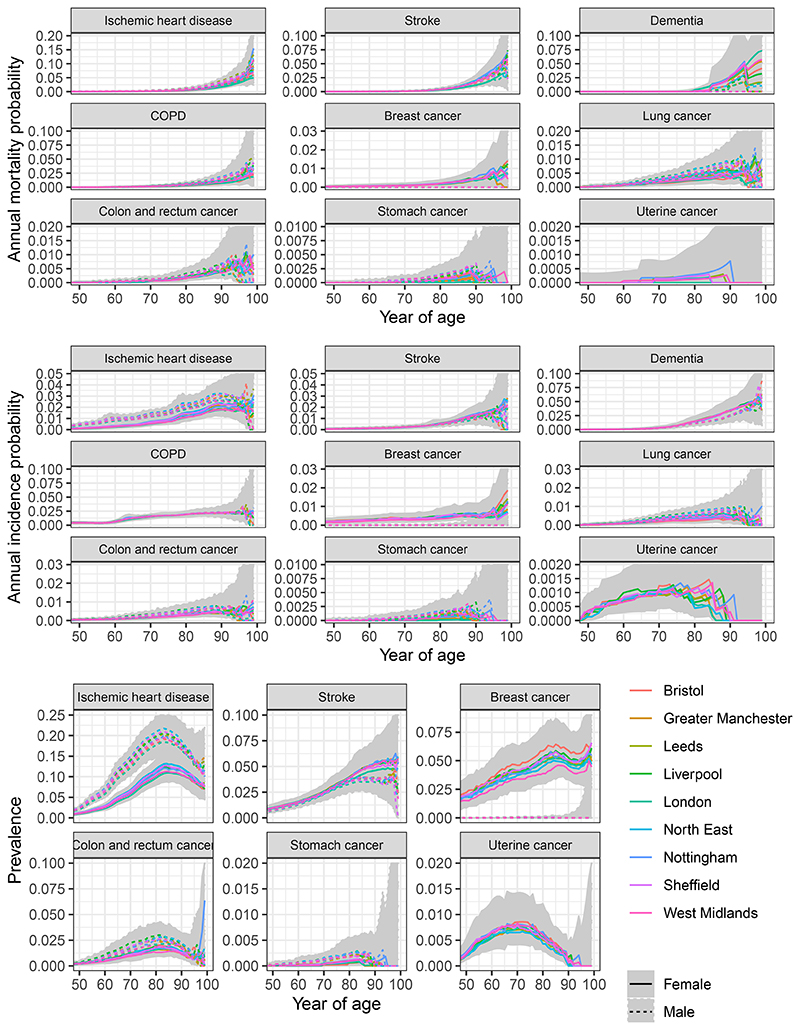
Mortality, incidence and prevalence by disease, age, gender, and city region in England, 2019. Estimates from the Global Burden of Disease, smoothly disaggregated from 5-year age groups. Shaded areas encompass the published 95% credible intervals from all city regions (some are truncated above).

Figure 2 illustrates these data, in the form of estimated probabilities and 95% credible intervals that are approximately equivalent to the numerators and denominators. This shows how the mortality, incidence and prevalence for each disease changes with age, for people over 50, compared between city regions and genders. Both the incidence and mortality are lowest for uterine cancer and stomach cancer. Differences between city regions are moderate compared to differences between ages. The extent of uncertainty tends to increase with age, and most estimates are highly uncertain beyond age 90.

### 3.2 Estimates from models fitted to areas independently

For each disease, city region and gender, the model of Section 2.3 was fitted to estimate the case fatality given the mortality, incidence and prevalence. Case fatality, incidence and remission (where included) were smoothed as functions of age (as in Section 2.4), while remission rates for diseases other than cancer were assumed to be zero. The baseline age *a_base_*, below which rates were assumed constant, was varied according to the disease and the data that were available for younger ages, with *a_base_* = 70 for dementia, uterine cancer and stomach cancer, 30 for ischemic heart disease and 50 for all other diseases. Case fatality rates were also constrained to be increasing with age for dementia, stomach cancer, lung cancer and uterine cancer. For uterine cancer and stomach cancer, city-region-specific estimates of case fatality could not be obtained due to the small numbers of deaths per area and age (75 deaths overall for uterine cancer, and less than around 10 cases of stomach cancer per year of age and city region) leading to difficulties with estimation. Instead, only national estimates were produced for these diseases. For a small subset of diseases and areas, the parameters λ_0_, determining the smoothness of the age-dependence, were fixed at their posterior modes (obtained from optimisation) to stabilise MCMC estimation.

**Figure 3:**
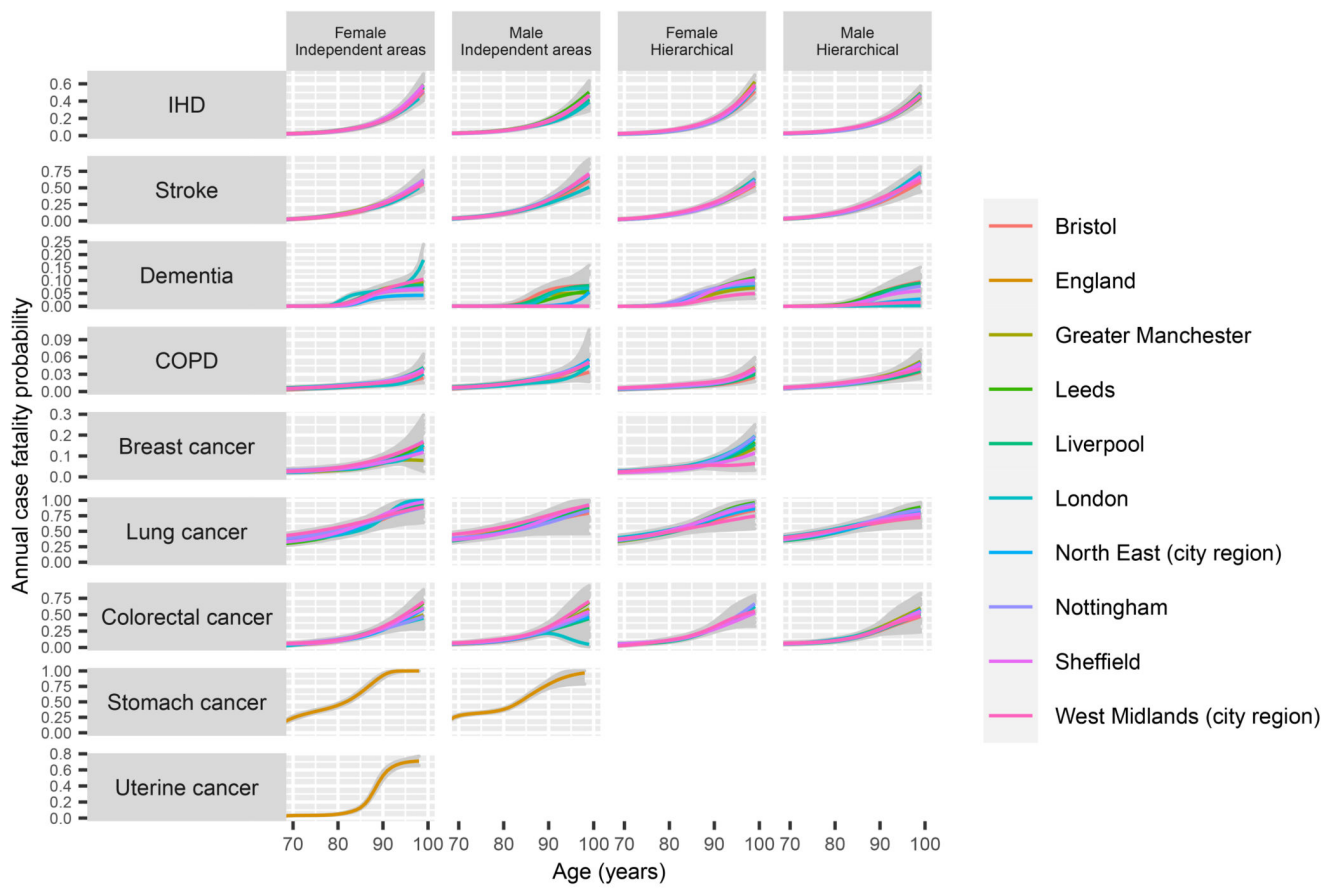
Estimates of the case fatality probability for chronic diseases, compared between men and women, and comparing hierarchical models with models that treat city regions independently. Each panel includes 9 lines denoting the posterior median from each of the 9 city regions, and the shaded area encompasses the 95% credible intervals from all city regions. For stomach and uterine cancer, only national estimates are produced from this model.

For each disease, the posterior median and 95% credible intervals for the annual case fatality probability, 1 – exp(–*i_a_*), obtained by MCMC under this model, are illustrated in the first two columns of Figure 3. Uncertainty is high beyond age 90 for most diseases, and the extent of between-area variability at younger ages is highest for lung cancer.

The approximation to the posterior around its mode is compared to the posterior from full MCMC in Appendix Figure 1, for case fatality estimates for multiple diseases, from women of a range of ages, in a single area. The intervals produced by normal approximation around the mode generally agree with the intervals from the quantiles of the MCMC sample, while the posterior mode and median are similar. This shows that a principled point estimate, together with a reasonable characterisation of the extent of uncertainty, can be produced without the expense of MCMC sampling (which took about 5 minutes per disease and patient group in this example, compared to 1 second for the approximation).

### 3.3 Hierarchical models

The variability between these area-specific estimates may be driven by noise, particularly where the uncertainty in the data is greatest for ages over 90 (Figures 2,3). In these cases, a hierarchical model can allow information to be shared between areas, without assuming the risks are equal between areas. The hierarchical model from Section 2.5 was implemented while also supplementing the data from 9 city regions of England with data from the rest of England. Since sufficient direct data on incidence are available for each area, the random effects model was only used for case fatality, rather than incidence. A mutually-exclusive set of 17 areas covering the whole of England was defined by (a) 9 city regions, (b) the regions not containing these city regions, and (c) the regions containing these city regions but with the city-region data excluded, using the lookup table at https://geoportal.statistics.gov.uk/datasets/0c3a9643cc7c4015bb80751aad1d2594/explore.

The hierarchical models were more difficult to develop, due to the complexity of the posterior distributions and the computational expense. For all diseases, stable estimation required the parameters λ_0_ and $\lambda_{0}^{\left(inc\right)}$, describing the smoothness of the rates as functions of age, to be fixed. These were set to plausible values determined from the non-hierarchical models. For uterine and stomach cancer, arbitrarily strong restrictions were required to enable between-area variations in case fatality to be estimated, specifically, an assumption that case fatality was a constant or log-linear function of age. For these less common diseases, we would judge that precisely capturing area-level differences is not essential for informing health impact models for physical activity interventions, therefore we present only country-level estimates.

In general, the hierarchical and non-hierarchical models gave substantively the same estimates, except for the oldest ages where the data are sparsest. At those ages, the variability between the area-specific estimates, and the extent of uncertainty, is less than under the model where areas are treated independently, since each area’s estimate is “shrunk” towards the data from other areas, illustrated in the third and fourth columns of Figure 3. The cross-validatory statistics Σ_*i*_*elpd_i_*, however, indicated that the non-hierarchical models had better predictive ability on the whole (Appendix Table 2).

**Figure 4:**
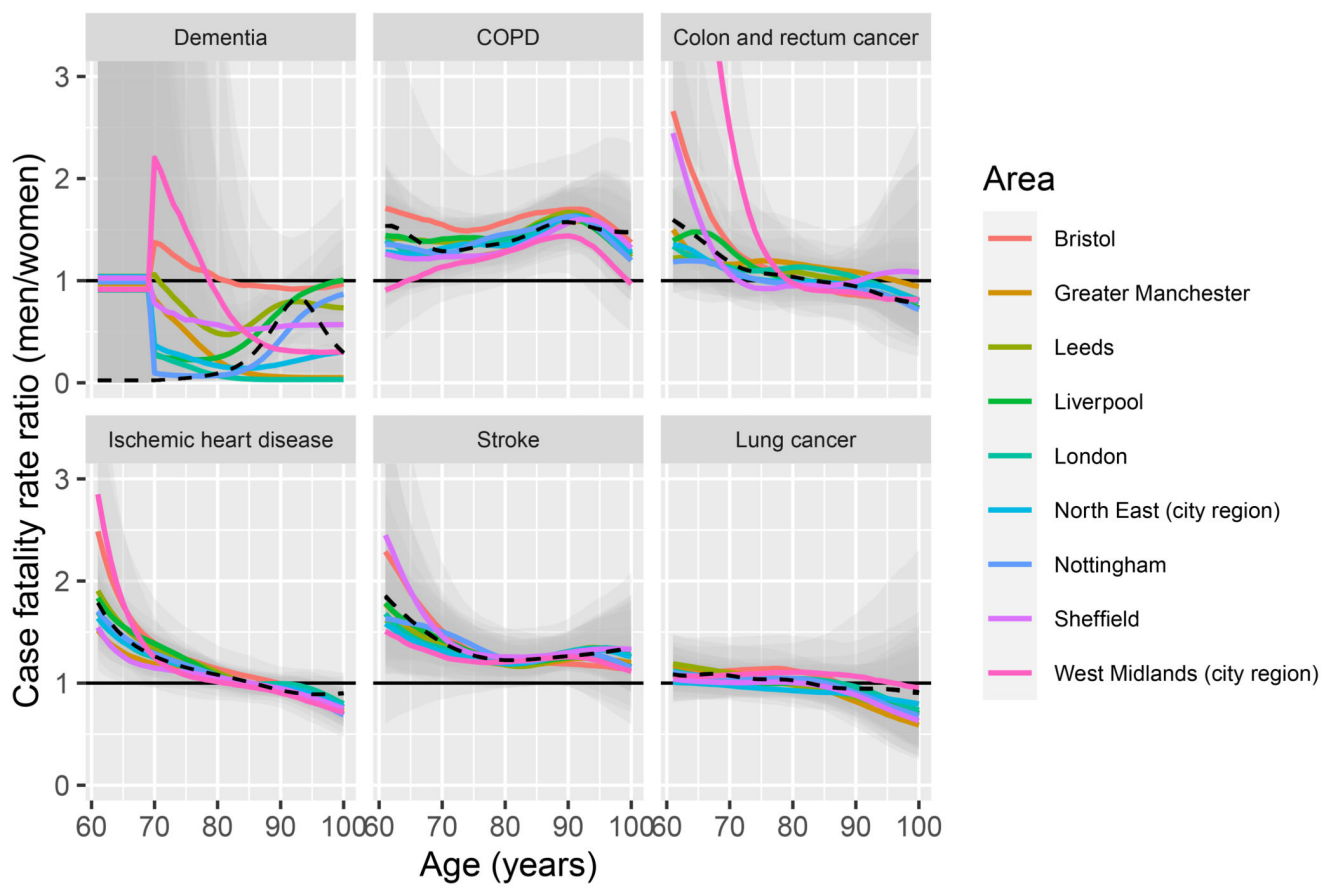
Ratio of case fatality rate between men and women, for seven diseases, by age and city regions of England. Posterior medians from the hierarchical model as coloured lines, with 95% credible intervals in faint grey. Posterior median from the hierarchical model with gender effect independent of area shown as a black dotted line. Horizontal axis restricted to exclude the estimates with large uncertainty.

Figure 4 illustrates the effect of gender. The ratio of case fatality rates between men and women, estimated from the hierarchical models, is shown as a function of age, by disease and area. For most diseases, the shape of the age-dependence of this gender effect generally appears similar between areas, with any differences in the estimated shape explainable by uncertainty from sparse data (e.g. colorectal cancer for around age 70 and younger). For dementia, there is a large variability between these estimates that is driven by the small numbers of deaths by gender and area, in particular for men. Therefore another set of hierarchical models was fitted, with the additional assumption that the relative case fatality between women and men was the same in every area (Section 2.6), i.e. area and gender effects are additive. The posterior median of the equivalent area-independent gender effect from this model is superimposed on Figure 4. For all diseases, apart from colorectal cancer and ischemic heart disease, the cross-validatory information criterion −2Σ_*i*_*elpd_i_* was lower for the model with additive gender and area effects (Appendix Table 2), indicating that it provided a better overall description of the data for that disease than the model where the gender effect interacts with the area effect. For dementia, the additive model appears to be under-smoothing the effect of gender around ages 70-75.

### 3.4 Time trends

The model with areas treated independently was extended to include time trends for ischemic heart disease. Including time trends in the hierarchical model was computationally infeasible. Estimates of trends over time in the incidence of myocardial infarction and subsequent case fatality (in terms of 30-day survival) are obtained from three sources: Scarborough et al. (2011) (for 1968–1998 in Oxfordshire, England), Smolina et al. (2012) (for 2002–2010 in England) and British Heart Foundation (2020) (for 2010–2019 incidence in the UK). These data are illustrated in Appendix Figure 2. No relevant data on case fatality after 2010 were found — instead the trend from 2003–2010 was assumed to continue from 2010 to 2019. Note that each source presents estimates by a different age grouping, leading to some inconsistency in the age-specific trends. Estimates from 5-year age groups are converted to single years of age by smoothing, the 1968 values are assumed to apply to previous years, and the 1998-2002 values are interpolated, to obtain a matrix describing the ratio of incidence (or case fatality) rates between each calendar year and the current year (2019). These ratios are used in the model from Section 2.7, assuming that they apply to the incidence and case fatality for all ischemic heart disease, and that the trends apply identically to all areas of England. To assess sensitivity to the assumption that the trend of reduction in case fatality beyond 2010 was the same as in the previous decade, we investigated an alternative scenario where the 2010 rate remained constant in subsequent years.

**Figure 5:**
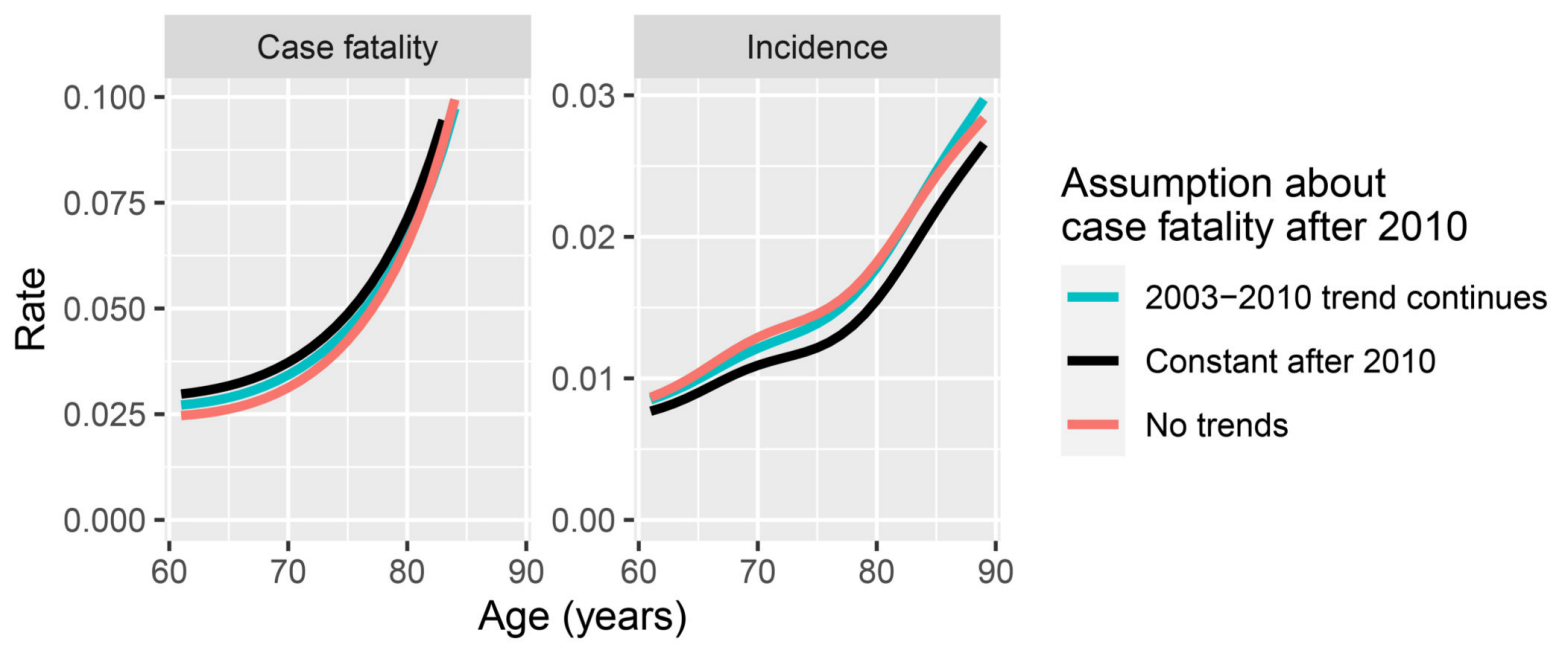
Estimates of case fatality and incidence for ischemic heart disease by age, for men in the Leeds city region in 2019. A model with no time trends in rates is compared with two models with time trends and different assumptions about case fatality after 2010.

Estimates of case fatality and incidence for ischemic heart disease in 2019, for an example area (Leeds city region), by age and gender, are plotted in Figure 5, comparing the model that ignored time trends with the models that included the estimates of past trends in these risks. Two assumptions about the trends in case fatality after 2010 are compared. Estimates of case fatality under age 80 are slightly higher, and estimates of incidence slightly lower, when these trend estimates are included. The differences are more substantial under the assumption where case fatality does not change after 2010.

Scarborough et al. (2016) estimated a similar size and direction of bias for estimates of IHD incidence from prevalence data, when the Dismod II model is used and the time trend is ignored. Note that our model extends the model in DisMod II to allow the time trend to vary with age. In general, it is difficult to predict how accounting for a specific past trend will change estimates of current incidence or case fatality rates from current mortality and prevalence data. The current disease outcomes depend on both the current incidence and case fatality and the current prevalence (equation 2) which depends on the risks in previous years for the people represented in the current data. If these people faced higher risks of both incidence and case fatality in the past, the effect on the current prevalence (and hence our estimates) is hard to predict. The model can enable sensitivity analysis in situations where the past trends and their influence are uncertain.

### 3.5 Data consistency and goodness of fit

Finally we show how the models can be checked against observed data, and show the influence of different data sources on the estimated rates. Again we select a single disease, area and gender for illustration. The model for ischemic heart disease with no time trends is compared against the model with time trends (assuming trends continue after 2010), for men in the Leeds city region. Additionally we fit two further models, with and without time trends, in which the incidence data were excluded, hence case fatality and incidence rates are inferred from current prevalence and mortality data alone.

The observed data (in the form of proportions) are compared against posterior mode estimates of the theoretical probabilites assumed (Section 2.3) to generate those data. Specifically we compare

- $y_{a}^{\left(inc\right)}/n_{a}^{\left(inc\right)}$ with estimates of the theoretical annual incidence probability 1 – *P*_*a*11_
- $y_{a}^{\left(prev\right)}/n_{a}^{\left(\text{prev }\right)}$ with estimates of the theoretical prevalence *π_α_*
- $y_{a}^{\left(mort\right)}/n_{a}^{\left(mort\right)}$ with estimates of the theoretical annual mortality probability *d_a_*

In the models with time trends, the theoretical probabilities are based on the 2019 rates, to match the year of the data, but here the year subscript in the notation of Section 2.7 is suppressed for clarity.

**Figure 6:**
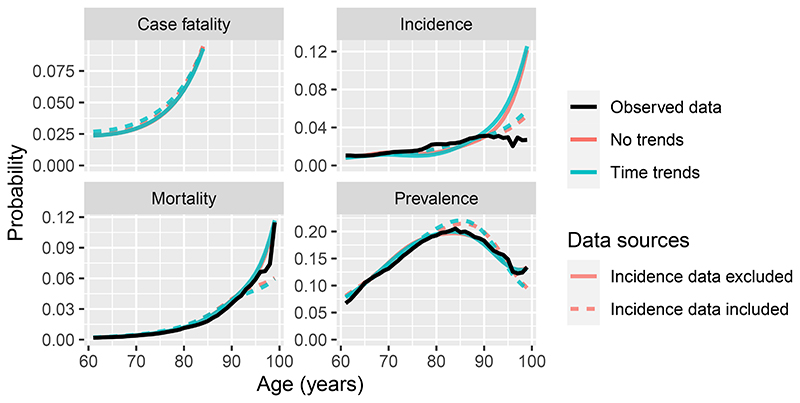
Estimated (posterior mode) case fatality, incidence, prevalence and mortality probabilities for ischemic heart disease for men in the Leeds city region, compared with corresponding observed proportions for all of these measures except case fatality, which is unobserved. A model with and without time trends is compared, and a model (without time trends) fitted to incidence, prevalence and mortality data is compared with a model fitted to just prevalence and mortality data (excluding incidence).

The estimates from the models that combine all three sources of evidence are shown as dotted lines in Figure 6. When the incidence data are included in the fit, there is some disagreement between the observed and fitted incidence data, and between the observed and fitted mortality and prevalence data beyond around age 80. When the incidence data are excluded, the fit to the incidence data is worse. This suggests that the incidence and prevalence data provide conflicting information about incidence rates. The estimates disagree even if a time trend is included, suggesting the inclusion of time trend data is insufficient to explain this conflict. This may either because the time trend data used here do not accurately represent the trends in risk experienced by the population behind the GBD data, or that the incidence data represent a different population from the prevalence and/or mortality data. The inferred case fatality rates only change moderately when the incidence data are excluded, however.

While the point estimates disagree slightly, the associated uncertainty (not plotted) is large enough to suggest that any conflicts are not statistically significant. This was tested formally by computing the conjugate posterior distribution for the probability *p* underlying each observed proportion, by combining, e.g. $y_{a}^{\left(inc\right)}∼Bin(n_{a}^{\left(inc\right)},p)$ with a Beta(0.5, 0.5) prior. The posterior probability *q* that *p* < *p*^(*full*)^ (where *p*^(*full*)^ is the comparable quantity from a synthesis of indirect evidence) is then computed to obtain a two-sided *conflict p-value* 2 min(*q*, 1 – *q*) (Presanis et al., 2013), which is < 0.05 in less than 5% of all cases, favouring the hypothesis that *p* = *p*^(*full*)^.

If conflict is a concern, then the incidence data could be excluded to ensure that the inferred prevalence and mortality match the corresponding observed data. Therefore, to maintain a transparent connection between the inferences and the data, while minimising assumptions about time trends and similarity between populations,the case fatality estimates could be taken from the prevalence and mortality data alone, while taking incidence estimates from the incidence data alone. Intuitively, current prevalence and mortality data are more informative about the current case fatality rate (which describes the risk of death for the population with prevalent disease) than current incidence rates. In Equation 2, *P*_*a*13_, the risk of both getting the disease and dying from it within a year, will generally be much smaller than the case fatality probability *P*_*a*23_, suggesting that the data on prevalence π_α_ and mortality *d_a_* are more important for learning *P*_*a*23_ than data on incidence.

Finally, the non-hierarchical models from Sections 3.2 were fitted again for all diseases with the incidence data excluded. (Without the direct data on incidence, the MCMC samplers for the hierarchical models failed to adapt and converge to the posterior distribution within a day of run time, even with hyperparameters fixed.). These were seen to fit the data slightly better on average than the models excluding incidence data, though both fitted adequately and the difference in their estimates was small (see Appendix Section E.1).

## 4 Discussion

This paper has provided more principled, transparent and flexible methodology, and accessible software, for estimating disease incidence and case fatality rates given indirect data. The methods were motivated by the requirements of multistate lifetable models to assess the health impacts of interventions or scenarios for prevention of chronic diseases. The methods may also be more widely useful for describing the burden of disease in settings where only indirect data are available, as has previously been done in the Global Burden of Disease project. We have illustrated a range of models of different complexity and computational expense. In principle, the Stan software that was used would enable variations and extensions of these models to be programmed in the same way, though in practice, more complex models may be more difficult to identify from the data. More complex models may also be more computationally intensive, and MCMC estimation for complex models can be particularly challenging where the data are weaker, as we found for the less common cancers. However, we have shown how optimisation can be used to provide point estimates of rates, and approximate credible intervals, without the expense of MCMC.

The required model complexity depends on the purpose of the model. For describing the burden of disease in different populations, it may be helpful to extend the model to allow more detail, e.g. to capture variations between subgroups other than by age and gender (using parametric assumptions as in Section 2.6), to describe spatial correlations between smaller areas, or to use covariate information to strengthen prediction at smaller areas. In contrast, if the ultimate aim is to estimate population health impacts of, e.g. changes in physical activity or diet, then simpler models will often be sufficient, since detail is only required if it would affect the estimates of impact. For example, for a rare disease, the information about case fatality will automatically be weaker, however impacts on a rare disease will only form a small part of the population impacts. Thus it may not be worth capturing variations more precisely by a more structured model, unless the specific impacts on that disease are of interest.

Ideally there would be no need for models, and instead, disease burden would be estimated from direct data. While uncertainty in our model-based estimates can be quantified, models rely on structural assumptions, hence these uncertainties are likely to be understated. These assumptions include the approximation of disease as a three-state Markov process, which may conceal variations between individuals and through time. For many diseases, there are risk prediction models (e.g. QRisk, Hippisley-Cox et al. (2017)) that give better descriptions of how incidence varies with individual characteristics, and these can be used as part of microsimulation models for estimating health impacts (Mytton et al., 2017). Case fatality, however, is less well understood. The Markov assumption, that case fatality does not depend on time since onset, is unrealistic for some diseases. Commonly it is higher soon after onset, when acute events, such as myocardial infarction, may have occurred. Case fatality rates are also affected by treatments and the presence of other diseases (multimorbidity). While violation of these assumptions is a concern if case fatality rates are of direct interest, it is less clear how much these assumptions would affect estimates of the impacts of prevention interventions. Interaction between diseases might be represented in a multi-state model by introducing “combined” disease states (Lauer et al., 2003), though the computational difficulty and data requirements would increase rapidly with the number of diseases. Another approach to health impact modelling is to disregard causes of death and model the all-cause mortality rate for people with each disease separately, see, e.g. Boshuizen et al. (2017).

In our application, we determined case fatality and incidence estimates, for several diseases assumed to be relevant to active transport, relating to city regions in England, to inform multistate health impact models. For ischemic heart disease, one of the diseases most affected by physical activity, we extended previous methods to show how including information about past trends in age-specific risks can have a substantial impact on estimates of current risks. We illustrated the uncertainty in estimates that arises from conflicting information in different sources of data, and uncertainty about past time trends. Note also that the Global Burden of Disease estimates that we used as data for our case study are likely themselves to have been derived from models, but the full details of how they are derived are unclear. These uncertainties show that better data is needed on case fatality rates for chronic diseases, with transparent information about how the data were generated. This would provide better information about disease burden, and enable better-informed estimates of the impacts of interventions to prevent disease.

## Supplementary Material

Supplementary File
