# Supplementary material for: Bayesian multistate modelling of incomplete chronic disease burden data
Source: J R Stat Soc Ser A Stat Soc. Author manuscript; Available in PMC 2023 Mar 6. (PMC7614284; doi:10.1093/jrsssa/qnac015)
Supplement: Supplementary File [file EMS158740-supplement-Supplementary_File.pdf]

Bayesian multistate modelling of incomplete chronic disease burden data: Appendices for online publication

Christopher Jackson, Belen Zapata-Diomedi, James Woodcock

A Transition probabilities in terms of rates

The disease model described in section 2.1 of the main manuscript is a continuous-time Markov model with the transition intensity matrix at age a years:

$$Q_a = \begin{pmatrix} -q_a & q_a & 0 \\ r_a & -(q_a + r_a) & f_a \\ 0 & 0 & 0 \end{pmatrix}$$

The corresponding *transition probability matrix* $P_a(t)$ is the matrix of probabilities $p_{a,j,k}(t)$ that a person in state j at an age of exactly a years is in state k at a point t years later. This is the solution to the Kolmogorov forward equation $\frac{d}{dt}P_a(t) = P_a(t)Q_a(t)$, with $P_a(0)$ equal to the identity matrix (see, e.g. Cox and Miller, 1977). For a person aged exactly a years, the annual transition probabilities $P_a = P_a(1)$ governing their state at age $a+1$ can be obtained as a closed-form function of the incidence q_a, case fatality f_a and remission r_a rates at that age, since these rates are assumed to be constant within a single year of age. As described in Barendregt et al. (2003), and dropping the a subscript for clarity, the j,k entries $p_{j,k}$ of this matrix are

$$\begin{aligned}
p_{1,1} &= (2(\nu - \omega)(f + r) + \nu(\kappa - \psi) + \omega(\kappa + \psi))/(2\kappa) \\
p_{1,2} &= q(\nu - \omega)/\kappa \\
p_{1,3} &= (-\psi(\nu - \omega) - \kappa(\nu + \omega))/(2\kappa) + 1 \\
p_{2,1} &= (\nu - \omega)r/\kappa \\
p_{2,2} &= -((2(f + r) - \psi)(\nu - \omega) - \kappa(\nu + \omega))/(2\kappa) \\
p_{2,3} &= ((\nu - \omega)(2f - \psi) - \kappa(\nu + \omega))/(2\kappa) + 1 \\
p_{1,3} &= 0 \\
p_{2,3} &= 0 \\
p_{3,3} &= 1
\end{aligned}$$

where state 1 is no disease, state 2 is disease, state 3 is death, $\psi = q+r+f$, $\kappa = \sqrt{q^2 + 2qr - 2qf + r^2 + 2fr + f^2}$, $\omega = \exp(-(\psi + \kappa)/2)$ and $\nu = \exp(-(\psi - \kappa)/2)$.

B Obtaining count data from published information

Disease epidemiology summaries might be published as proportions rather than counts. For example, prevalence may be published as an estimated proportion of people with the disease $p = \hat{p}_a^{(prev)}$. To convert this into a count r and denominator n, as required for the model in Section 2.3 of the main manuscript, additional information is required (dropping the age subscript in this section for clarity). Most simply, if the population size n that informed the estimate is published, or can be confidently estimated, then we determine the count as $r = \hat{p}n$.

If the population size is unknown, but a interval estimate $(\hat{p}^{(L)}, \hat{p}^{(U)})$ (95%, say) is published that represents uncertainty about the point estimate $\hat{p}$, then this information can be converted to an implicit numerator r and denominator n with a Bayesian technique used in expert elicitation (O'Hagan et al., 2006). This assumes that the point and interval estimates are summaries of a Beta posterior distribution which has been obtained by combining a vague prior with an observation of r events occurring out of a sample of n individuals. For example, with

a vague $Beta(0,0)$ prior (which is uniform on the logit scale) the posterior is $Beta(r, n-r)$. We can then search for the best-fitting $Beta(\alpha, \beta)$ distribution which has median $\hat{p}$ and (2.5,97.5) quantiles $(\hat{p}^{(L)}, \hat{p}^{(U)})$, and set $r = \alpha, n = \alpha + \beta$. This is done by minimising the following expression with respect to α and β using numerical optimisation. This expression is the sum of squared errors between the published quantiles and the theoretical quantiles based on the Beta distribution,

$$\left(F(\hat{p}^{(L)}|\alpha,\beta) - 0.025\right)^2 + \left(F(\hat{p}|\alpha,\beta) - 0.5\right)^2 + \left(F(\hat{p}^{(U)}|\alpha,\beta) - 0.975\right)^2$$

where F is the cumulative distribution function of the Beta(α, β).

If we also suspected that one of the data sources may be biased, but were unsure about the direction of bias, we could downweight that data source by multiplying both r and n by the same amount and rounding, e.g. by 0.5 if we wanted to give a data source half its original weight.

C Handling data coarsened by age groups

A further complication of typical chronic disease burden data is that it may be published as estimates by broad age groups, e.g. 5 or 10-year ranges, possibly of unequal widths, while the multistate disease model of Section 2.1 of the main manuscript requires data for each year of age. Flaxman et al. (2015) discuss a variety of methods for modelling rates published on unequally-spaced age groups, but acknowledge computational or statistical limitations with them all. In DisMod II, for example, rates for coarse age groups can be converted to smooth functions of age by polynomial or spline interpolation, however that is not guaranteed to preserve the underlying information that the estimates were obtained from.

Instead, we use *smooth temporal disaggregation*, applied to the count data, rather than the underlying rates. For example, given a published prevalence count of $\sum_{a=51}^{55} {y_a}^{(prev)}$ aggregated over ages 51–55, counts per year of age could be produced naively by dividing the 5-year total by 5. However, *smooth* disaggregation methods produce more realistic estimates of ${y_a}^{(prev)}$ that form a smooth function of a over all age intervals, while preserving the sum within each age interval to exactly equal the published count. A variety of methods for doing this efficiently are implemented in the `tempdisagg` R package (Sax and Steiner, 2013). The method we use is based on a linear regression for the grouped data as a function of the age group, with the year-specific data assumed to be correlated through a first-order autoregressive model (Chow and Lin, 1971).

D Spline basis

Incidence and case fatality rates are modelled as spline functions of age (Section 2.4 of the main manuscript). A thorough review of different spline-based and other generalised additive models is given by Wood (2017). We use the *thin plate regression spline* recommended there due to its theoretical optimality properties, and because it does not require a choice of knots. However, different ways of constructing splines, including cubic splines, gave similar results in our applications.

A *thin plate spline* basis, for a regression of an outcome y_i on a predictor x_i based on observations $i = 1, \ldots, n$, is derived as the function f that minimises

$$\sum_{i=1}^{n} (y_i - f(x_i))^2 + \lambda_0 \int \left(\frac{\partial^2 f}{\partial x^2}\right)^2 dx$$

the average (squared) difference between the data and the fitted values, penalised by the average smoothness of f, as measured by its (squared) second derivative. The minimising function takes the form of a cubic smoothing spline which directly interpolates each of the x_i. Since this function has as many parameters as there are data points, a lower-dimension basis, termed the *thin plate regression spline*, is obtained by truncating the space that the basis spans. This approximated basis is defined by the eigenvectors associated with the K largest eigenvalues of the full basis matrix.

Therefore the number of parameters to be estimated is not defined by the number and location of "knots", as in a cubic regression spline, but by the degree K of approximation. The penalty λ_0 governs the overall smoothness, and is given a prior and estimated as part of the Bayesian procedure, as we describe in Section 2.4 of the main manuscript. Further details of this procedure are given in Wood (2017), pp215–218. and Wood (2003).

E The ‘disbayes’ R package

An R package, `disbayes`, was developed, that implements all the models used in this paper. It is available from `https://chjackson.github.io/disbayes`. A brief example of its use and a description of its features is given here, but full documentation is available as R help pages for the functions in the package, and in a “vignette” containing worked examples.

E.1 Non-hierarchical models

The user must supply a data frame with one row for each year of age (beginning at year 0 but ending at any year), for a homogenous population (e.g. as defined by a single area and gender). The columns contain estimates of at least mortality and at least one of incidence or prevalence, and optionally also remission. The recommended form is as numerators and denominators, as described in Section 2.3 of the main manuscript, so that rows of the data might look like this:

```
    age gender area  inc_num inc_denom prev_num prev_denom mort_num mort_denom ...
1    60 Male   Leeds      55      5252      180       2636       20      12386
2    61 Male   Leeds      64      6157      168       2217       21      11978
3    62 Male   Leeds      68      6437      163       1901       22      11700
4    63 Male   Leeds      65      6095      163       1686       24      11553 ...
```

Alternatively, point and interval estimates, or point estimates and denominators, can be provided. The heuristic explained in Appendix Section B is used internally to convert credible intervals to numerators and denominators. The important requirement is that as well as an estimate, some indication of sampling uncertainty must be provided, either through a denominator or a credible interval. This enables formal statistical inference.

If data are available as coarsened age intervals, this must first be converted to counts per year of age, e.g. using the methods discussed in Appendix Section C. A worked example of this is provided in the package vignette.

The model in Sections 2.3–2.4 of the main manuscript is then fitted with a command such as the following

```
db <- disbayes(data = dat,
               inc_num = "inc_num", inc_denom = "inc_denom",
               mort_num = "mort_num", mort_denom = "mort_denom",
               prev_num = "prev_num", prev_denom = "prev_denom",
               eqage = 30)
summ <- tidy(db)
```

where the argument `dat` gives the name of the data frame, the following six arguments name the variables in that data giving numerators and denominators for incidence, mortality and prevalence, and `eqage=30` specifies that case fatalities are assumed to be equal for all ages below 30 (Section 2.4 of the main manuscript).

Additional options to the `disbayes` function include:

- `cf_model`: model for case fatality as a function of age. By default, a smooth function of age is used, `(cf_model="smooth")`, with a shape that is unrestricted except through `eqage`. Alternative options include a smooth increasing function `("increasing")`, a model where rates are constant with age (`"const"`), or unrestricted rates that are estimated independently for each age (`"indep"`). Similar options are available to specify the model for incidence or remission rates.

- `method`: The algorithm used to fit the model. The default `"opt"` uses optimisation to find the posterior mode, and obtains posterior credible intervals through a normal approximation. The alternative `"mcmc"` uses MCMC sampling from the full posterior, which is more accurate, but an order of magnitude slower. `rstan`’s variational Bayes methods can also be used, and may give a more accurate approximation to the posterior than `method="opt"` for not much more expense, but they have not been investigated in detail for this class of models.

- Options to control sampling or optimisation can be passed through `disbayes` to the underlying functions used by `rstan`.

- Options are available to specify the parameters of all prior distributions.

- `cf_trend`, `inc_trend`: matrices of constants describing trends through time in rates, in the form of the ratio of case fatality (or incidence) between previous years and the year of the data, by year of age.

- `disbayes` can also return leave-one-out cross-validation statistics that describe how well each observation in the dataset would be predicted if the model were fitted to the remainder of the data, defined and computed using the methods of Vehtari et al. (2017, 2020).

- `hp_fixed`. Sometimes there may be difficulties in obtaining the posterior distribution of the "hyperparameters" $\lambda_0, \lambda_0^{(inc)}$ that determine the smoothness of the function relating case fatality or incidence rates to age. In those cases, the λ_0 can be fixed at values supplied in this argument, obtained, e.g. from an estimate in a similar dataset, or from the posterior mode if that can be determined. Values of around 1–5 were estimated in the applications in the paper. Higher values give more flexible curves, and values close to zero approach a linear function for the log rate with age.

The object `summ` then contains point and interval estimates for all estimated quantities in the model as a "tidy" data frame, a form convenient for processing and plotting, e.g. by using the `dplyr` and `ggplot2` packages. For example, to extract from this data frame the posterior mode and approximate 95% credible limits for the case fatality rates for people aged from 61 to 65,

```
> library(dplyr)
> summ %>%
>    filter(var=="cf", between(age, 61, 65)) %>%
>    select(age, mode, "2.5%", "97.5%")
  age       mode       2.5%      97.5%
2  61 0.01480355 0.01234622 0.01738378
3  62 0.01507347 0.01263041 0.01766254
4  63 0.01535594 0.01296970 0.01803932
5  64 0.01565776 0.01337025 0.01833570
6  65 0.01598639 0.01373186 0.01868637
```

The variables that can be extracted from this data frame are documented in `help(tidy.disbayes)`.

E.2 Hierarchical models

The function `disbayes_hier` can be used in a similar fashion to fit the hierarchical models of Sections 2.5–2.6 in the main manuscript. Case fatality rates can have random intercepts and slopes, but random effects are not currently supported for incidence or remission. The dataset is supplied in the same form as above, but should describe data from multiple areas. For example, if there are 100 years of age, and 10 areas, then the data should contain 1000 rows giving disease outcomes for each area and age. Arguments to the `disbayes_hier` function include:

- `group`: name of the variable in the data indicating the area or other group that the estimate was obtained from.

- `gender`: name of the variable indicating gender (or other binary classification). This should be omitted if modelling a single gender. If it is supplied, then gender and area effects are assumed to be additive, as in Section 2.6, and the data should contain, e.g. 2000 rows if there are 100 ages and 10 areas.

- `hp_fixed`. A list of hyperparameters that should be fixed at supplied constants, or at their posterior modes. These might include the smoothness parameters λ_0 or the standard deviations of random effects. This might be used if there is difficulty sampling from a full hierarchical model that represents the posterior distributions of these parameters.

E.3 Summary of packages

The following table briefly summarises the differences between three alternative packages that are available for estimating the rates of the three-state disease model given indirect data on at least mortality, prevalence and/or incidence. The main manuscript of this paper explains these differences in more technical detail. A wide variety of useful models can be specified in any of these packages.

It is assumed that the methods described in detail in Flaxman et al. (2015) are those that are implemented in DisMod-MR, though the documentation included with the software itself is sparse. Note also that DisMod-MR has more features for modelling *direct* data such as prevalence data, including regression models to explain heterogeneity in disease burden between areas in terms of covariates. DisMod II also has an extensive graphical user interface for interactive data management and graphics, however we would argue that due to their "scriptable" nature, languages such as R or Python are preferred for reproducible research.

	DisMod II	DisMod-MR	`disbayes`
Statistical principles	Maximum likelihood	Bayesian	Bayesian
Age dependence of rates	Informal smoothing	Formal, fixed-knot penalised linear spline	Formal, more flexible penalised spline
Between-area variations in rates	Areas independent	Empirical Bayes	Fully-hierarchical or empirical Bayes
Time trends in rates	Age-common	None	Age-dependent
Regression models	No	Yes	No
Computational method	Optimisation (fast)	Metropolis MCMC (intensive)	Optimisation or Hamiltonian MCMC
Interface	Windows	Python	R

Table 1: Summary of characteristics of three alternative programs for multistate disease modelling from indirect data

F Supplementary data and results

F.1 Discrete-time estimation of case fatality

Estimates of case fatality can also be produced by dividing mortality by prevalence. This is a simple alternative to the full Bayesian model presented in the paper, but relies on stronger assumptions. We illustrate this in two ways. Firstly, under our model, if we also assume that a person cannot get a disease and die from it within the same year, this gives $P_{a13} = 0$, and Equation 2 becomes $d_a = P_{a23}\pi_a$. Therefore if we have estimates of the mortality *risk* d_a and the prevalence π_a, we can then estimate the annual case fatality risk P_{a23} as d_a/π_a.

Alternatively, without reference to a Markov model, a mortality *rate* is typically defined as $\lambda = E(Y)/N_{pop}$, where Y is the number of deaths from the disease in a population, and N_{pop} is the total person-years of follow-up over which the deaths were observed. Similarly, the case fatality rate is $f = E(Y)/N_{dis}$ where N_{dis} is the person-years of follow up for people with the disease. Therefore $f = \lambda/\pi$, where $\pi = N_{dis}/N_{pop}$. So if π is taken from an estimate of disease prevalence, and if this is assumed to be constant over the period of follow-up, we can estimate the case fatality rate by dividing an estimate of mortality by π. Note this assumption is not made in our Markov model.

Estimates of case fatality rates produced in this way (deemed "discrete-time" estimates) from for women in one city region (Leeds), and seven selected diseases, are compared with the full Bayesian estimates in Figure 1. The discrete-time estimates agree with the Bayesian posterior medians in many cases, though are noisy and disagree in some situations, particularly for the oldest ages where the data are weaker. The Bayesian estimates are more plausibly smooth as functions of age.

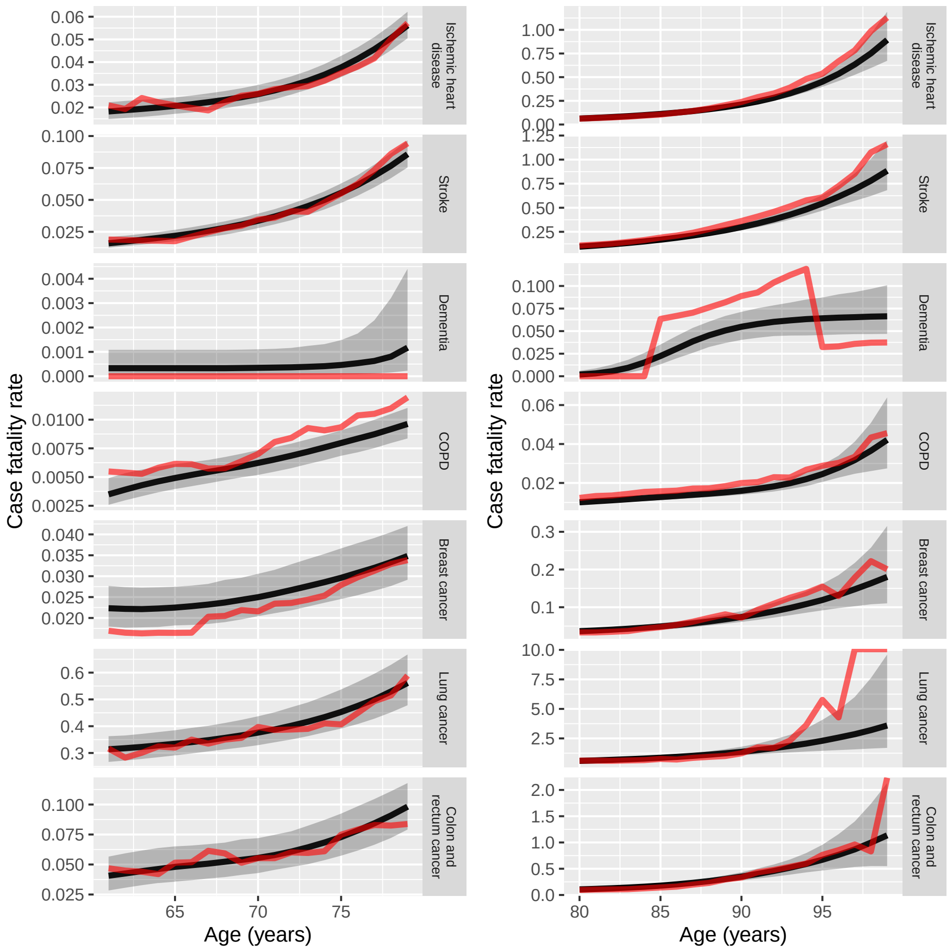

Figure 1: Discrete-time estimates of case fatality rates from mortality and prevalence (red lines), for women in Leeds, compared with medians and 95% credible intervals from the full Bayesian model. The horizontal axis of age is split into two ranges (50-80 and 80-100) to show the contrasts more clearly under the different scales.

F.2 Comparison of MCMC and optimisation

For each of the city region-specific models illustrated in Figure 3 of the main manuscript, we compare how well the posterior approximation obtained by optimisation (main manuscript Section 2.9) agrees with the results obtained from MCMC sampling.

In each case, optimisation took about one second. MCMC sampling, with four parallel chains of 1000 iterations, took about six minutes. The optimisation method produces the exact posterior mode, however, the posterior median and the credible intervals are produced by a normal approximation. We judge accuracy in two ways.

(a) the difference between the two estimates of the median case fatality (a measure of absolute bias).

(b) the relative percentage bias in the width of the 95% credible interval for case fatality: a measure of how well uncertainty is quantified. This is defined as $100(w_{opt} - w_{mcmc})/w_{mcmc}$, where w_{opt} and w_{mcmc} are the interval widths under the optimisation and MCMC methods respectively.

These measures are plotted against the absolute number of deaths in the data that informed each estimate (Figure 2, left) and against age (Figure 2, right). A specific example of the estimates produced for a single gender and area, comparing different diseases, is shown in Figure 3.

The approximate method consistently underestimates the extent of uncertainty (Figure 2, lower panels), which is a known property of variance estimators based on asymptotic normality (the "Cramér-Rao lower bound"). Relatedly, this method generally becomes more accurate when the estimate is based on a larger amount of data (Figure 2, left), both in terms of the bias of the median (upper left), and of the interval width (lower left).

The biases in the estimates of the medians are more consequential for higher ages, for which case fatality is higher (Figure 2, upper right, and Figure 3). This underlines the importance of using the posterior mode as the point estimate if the optimisation method is used, rather than the median. However for different age ranges, the extent to which the optimisation method underestimates uncertainty does not vary so much (lower right).

In summary, the optimisation method is orders of magnitude faster than MCMC, but while valid point estimates can be produced with this method, caution is required when using it to quantify uncertainty around these estimates, particularly if the data are weak.

F.3 Cross-validatory model comparison

For each of the outcomes for which we had area-specific data, that is, mortality, incidence and prevalence, the observation-specific expected log predictive density $elpd_w$ was computed via the Pareto-smoothed importance sampling method of Vehtari et al. (2017). This did not always give reliable estimates, since the importance sampling weights sometimes had an excessively high variance, as indicated by an estimated shape parameter $\hat{k} > 0.7$ for the generalized Pareto approximation to the weights. This happens when the full posterior has a much higher variance than the posterior after leaving out one observation, due to the left-out observation being particularly influential, in which case the full posterior is an unreliable basis for an importance sampling approximation.

In our models, $\hat{k} < 0.7$ consistently held for predictions of mortality between ages 50 and 90, but not for predictions of incidence or prevalence. Since the main goal of the models is to predict case fatality for chronic diseases within these ages, we judge this to be an adequate basis for comparing the non-hierarchical model with the hierarchical models with and without the additive effect of gender. Table 2 shows the results as differences in "leave-one-out information criteria" ($LOOIC = -2\sum_w elpd_w$) relative to the non-hierarchical model. The non-hierarchical models are preferred in most cases, except for COPD, where the additive hierarchical model is preferred, since the relative risk between men and women is similar between areas (Figure 4 in the main manuscript). The hierarchical model was also preferred for ischemic heart disease, though note that this model choice is of fairly minor importance, since the estimated case fatality probabilities differed by less than 0.01 between the models in over 90% of ages and areas.

Importance weights could not be computed accurately for the model without the incidence data (which is essentially based on a smaller dataset, hence more data-points are judged to be influential). Checks of agreement of estimates with data are more helpful for comparing the models with and without the incidence data, as illustrated in Section 3.5 of the main manuscript. Data as in Figure 6 of the main manuscript can be summarised numerically for all diseases and areas, e.g. the mean absolute difference between the observed mortality proportions and fitted mortality probabilities is 0.0007 for the model with incidence data, and by 0.0002 for the model excluding incidence data. Therefore, while excluding incidence data gives a slightly better fit, the agreement with the data is good for both models. Estimates of the case fatality probability under these two models differ by less than 0.01 for 90% of the age, area, gender and disease-specific estimates – therefore on the whole, these models do not severely disagree with each other.

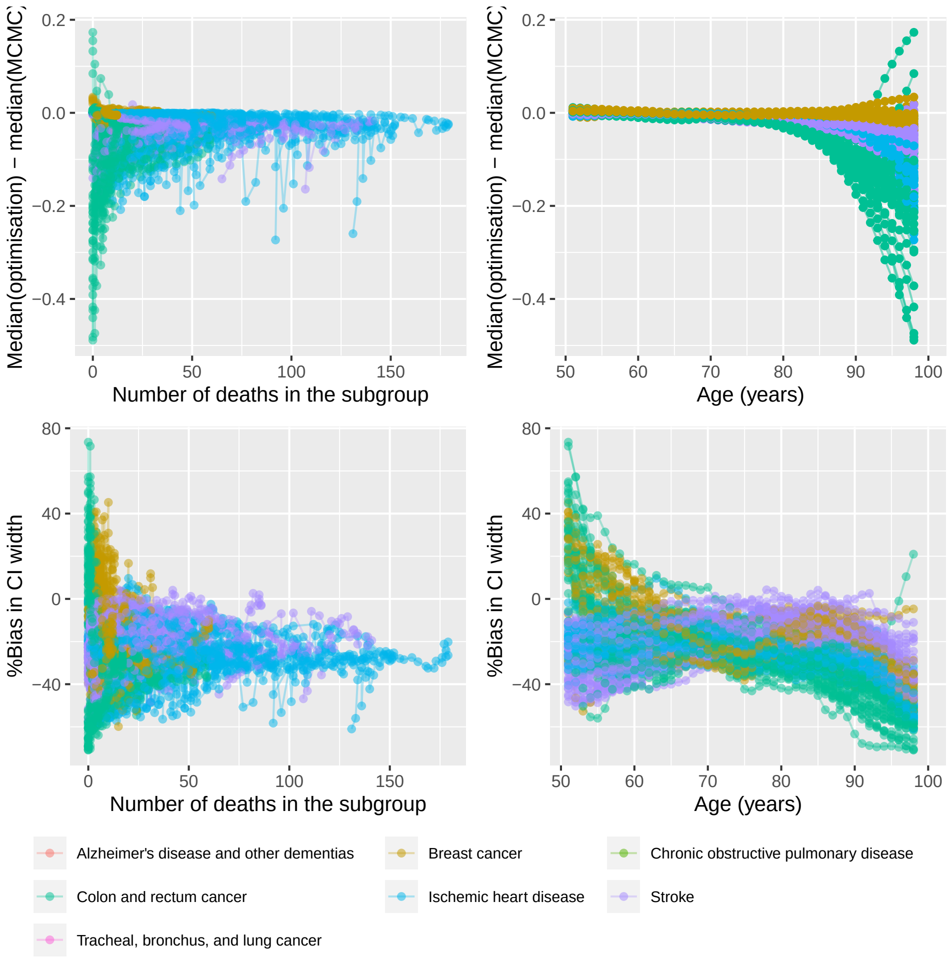

Figure 2: Accuracy of the estimates of the posterior median case fatality rate, and the width of the 95% credible interval (CI), from the optimisation-based posterior approximation method, compared to the MCMC method. Each point is a comparison between these two methods, for a different year of age, gender, disease and area

References

Barendregt, J. J., Van Oortmarssen, G. J., Vos, T. and Murray, C. J. L. (2003) A generic model for the assessment of disease epidemiology: the computational basis of DisMod II. *Population Health Metrics*, **1**, 4.

British Heart Foundation (2020) *Heart and Circulatory Disease Statistics 2020*. British Heart Foundation.

Chow, G. C. and Lin, A.-l. (1971) Best linear unbiased interpolation, distribution, and extrapolation of time series by related series. *The Review of Economics and Statistics*, 372–375.

Cox, D. R. and Miller, H. D. (1977) *The Theory of Stochastic Processes*, vol. 134. CRC Press.

Flaxman, A. D., Vos, T. and Murray, C. J. L. (2015) *An Integrative Metaregression Framework for Descriptive Epidemiology*. University of Washington Press.

O'Hagan, A., Buck, C. E., Daneshkhah, A., Eiser, J. R., Garthwaite, P. H., Jenkinson, D. J., Oakley, J. E. and Rakow, T. (2006) *Uncertain Judgements: Eliciting Experts' Probabilities*. John Wiley & Sons.

Sax, C. and Steiner, P. (2013) Temporal disaggregation of time series. *The R Journal*, **5**, 80–87. URL: `https://doi.org/10.32614/RJ-2013-028`.

Scarborough, P., Wickramasinghe, K., Bhatnagar, P. and Rayner, M. (2011) *Trends in coronary heart disease, 1961-2001*. British Heart Foundation.

Smolina, K., Wright, F. L., Rayner, M. and Goldacre, M. J. (2012) Determinants of the decline in mortality from acute myocardial infarction in England between 2002 and 2010: linked national database study. *BMJ*, **344**.

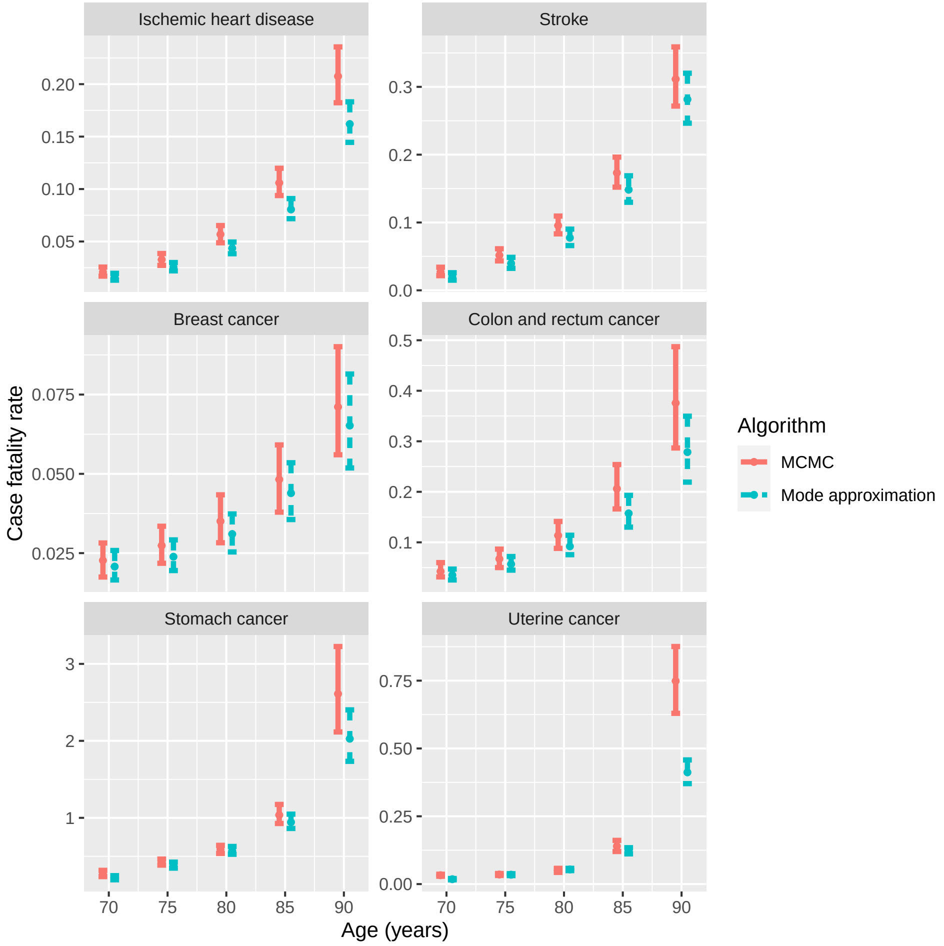

Figure 3: Case fatality probabilities for chronic diseases for women in Bristol (or, for uterine cancer and stomach cancer, in England), comparing posterior median and 95% credible intervals obtained from a MCMC sample with posterior mode and 95% credible intervals obtained from a normal approximation around the mode.

Vehtari, A., Gabry, J., Magnusson, M., Yao, Y., Bürkner, P.-C., Paananen, T. and Gelman, A. (2020) loo: Efficient leave-one-out cross-validation and WAIC for Bayesian models. R package version 2.4.1. URL: `https://mc-stan.org/loo/`.

Vehtari, A., Gelman, A. and Gabry, J. (2017) Practical Bayesian model evaluation using leave-one-out cross-validation and WAIC. *Statistics and Computing*, **27**, 1413–1432.

Wood, S. N. (2003) Thin plate regression splines. *Journal of the Royal Statistical Society: Series B (Statistical Methodology)*, **65**, 95–114.

— (2017) *Generalized Additive Models: an Introduction with R*. CRC, 2nd edn.

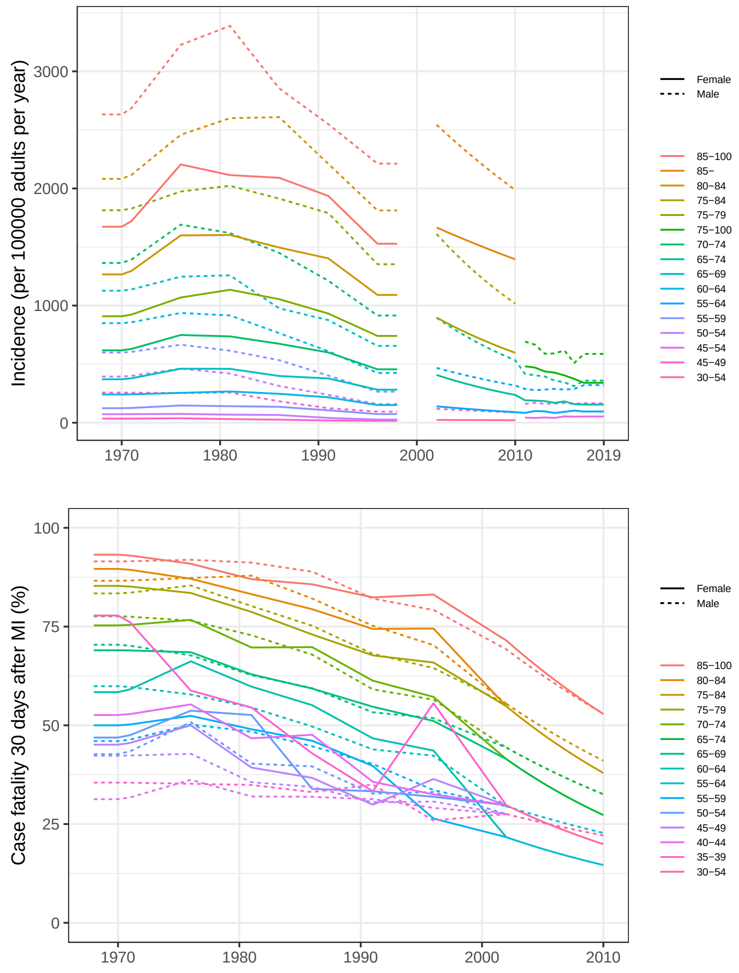

Figure 4: Estimated trends through time in the incidence (top) and case fatality (bottom) of myocardial infarction, by gender and age group (from Scarborough et al., 2011; Smolina et al., 2012; British Heart Foundation, 2020) (values in identical age groups are interpolated).

	Non-hierarchical model	Hierarchical models	
		Interaction	Additive
Ischemic heart disease	0	-34	7
Stroke	0	160	127
Dementia	0	124	108
COPD	0	23	-85
Breast cancer	0	-2	
Lung cancer	0	22	17
Colon and rectum cancer	0	4	17

Table 2: Leave-one-out cross validatory criteria comparing predictive ability of three models m: a non-hierarchical model $m = 1$ and two hierarchical models where the effects of gender and area either interact $(m = 2)$ or are additive $(m = 3)$ (or, for breast cancer, including only one gender). Predictive ability is judged against the mortality data between the ages of 50 and 90. The value of the criterion is presented as $LOOIC_m - LOOIC_1$, where $LOOIC_m = -2\sum_i elpd_{im}$, so that lower values are preferred, and values of 0 are shown for model $m = 1$. The non-hierarchical model is preferred in most cases.
